# γδ T Cells in the Tumor Microenvironment—Interactions With Other Immune Cells

**DOI:** 10.3389/fimmu.2022.894315

**Published:** 2022-07-11

**Authors:** Kok Fei Chan, Jessica Da Gama Duarte, Simone Ostrouska, Andreas Behren

**Affiliations:** ^1^ Olivia Newton-John Cancer Research Institute, and School of Cancer Medicine, La Trobe University, Heidelberg, VIC, Australia; ^2^ Department of Medicine, University of Melbourne, Parkville, VIC, Australia

**Keywords:** γδ T cells, αβ T cells, B cells, dendritic cells, macrophages, monocytes, natural killer cells, neutrophils

## Abstract

A growing number of studies have shown that γδ T cells play a pivotal role in mediating the clearance of tumors and pathogen-infected cells with their potent cytotoxic, cytolytic, and unique immune-modulating functions. Unlike the more abundant αβ T cells, γδ T cells can recognize a broad range of tumors and infected cells without the requirement of antigen presentation *via* major histocompatibility complex (MHC) molecules. Our group has recently demonstrated parts of the mechanisms of T-cell receptor (TCR)-dependent activation of Vγ9Vδ2^+^ T cells by tumors following the presentation of phosphoantigens, intermediates of the mevalonate pathway. This process is mediated through the B7 immunoglobulin family-like butyrophilin 2A1 (BTN2A1) and BTN3A1 complexes. Such recognition results in activation, a robust immunosurveillance process, and elicits rapid γδ T-cell immune responses. These include targeted cell killing, and the ability to produce copious quantities of cytokines and chemokines to exert immune-modulating properties and to interact with other immune cells. This immune cell network includes αβ T cells, B cells, dendritic cells, macrophages, monocytes, natural killer cells, and neutrophils, hence heavily influencing the outcome of immune responses. This key role in orchestrating immune cells and their natural tropism for tumor microenvironment makes γδ T cells an attractive target for cancer immunotherapy. Here, we review the current understanding of these important interactions and highlight the implications of the crosstalk between γδ T cells and other immune cells in the context of anti-tumor immunity.

## Introduction

For the past 37 years, since the first isolation of the TCR γ gene segment (1, 2), the knowledge accumulated about the γδ T-cell lineage has grown exponentially and received strong clinical interest, especially for cancer immunotherapy development (3–15). Similar to the other two lineages of lymphocytes in the jawed vertebrates that utilize somatically recombined receptors for immunosurveillance (B cells and αβ T cells) (16), TCR heterodimers of γδ T cells are generated through somatic rearrangements of genes encoding for TCR δ chain variable (V), diversity (D), joining (J), and constant (C) gene segments, and TCR γ chain V, J, and C gene segments at the thymus (17, 18). Hypothetically, such diverse gene rearrangements can result in a total of 10^17^ possible distinct γδ TCRs (19). Despite the diverse theoretical γδ TCR repertoire, human γδ T cells can be classified into two major subsets according to their TCR Vδ chain usage: Vδ2^+^ populations that are usually paired with Vγ9 chain, and Vδ2^−^ populations with diversified Vγ chain usage (6, 20). Among all 8 TCR Vδ gene segments, Vδ1, Vδ2, and Vδ3 are three commonly used segments for δ chain rearrangement (21, 22).

**Figure 1:**
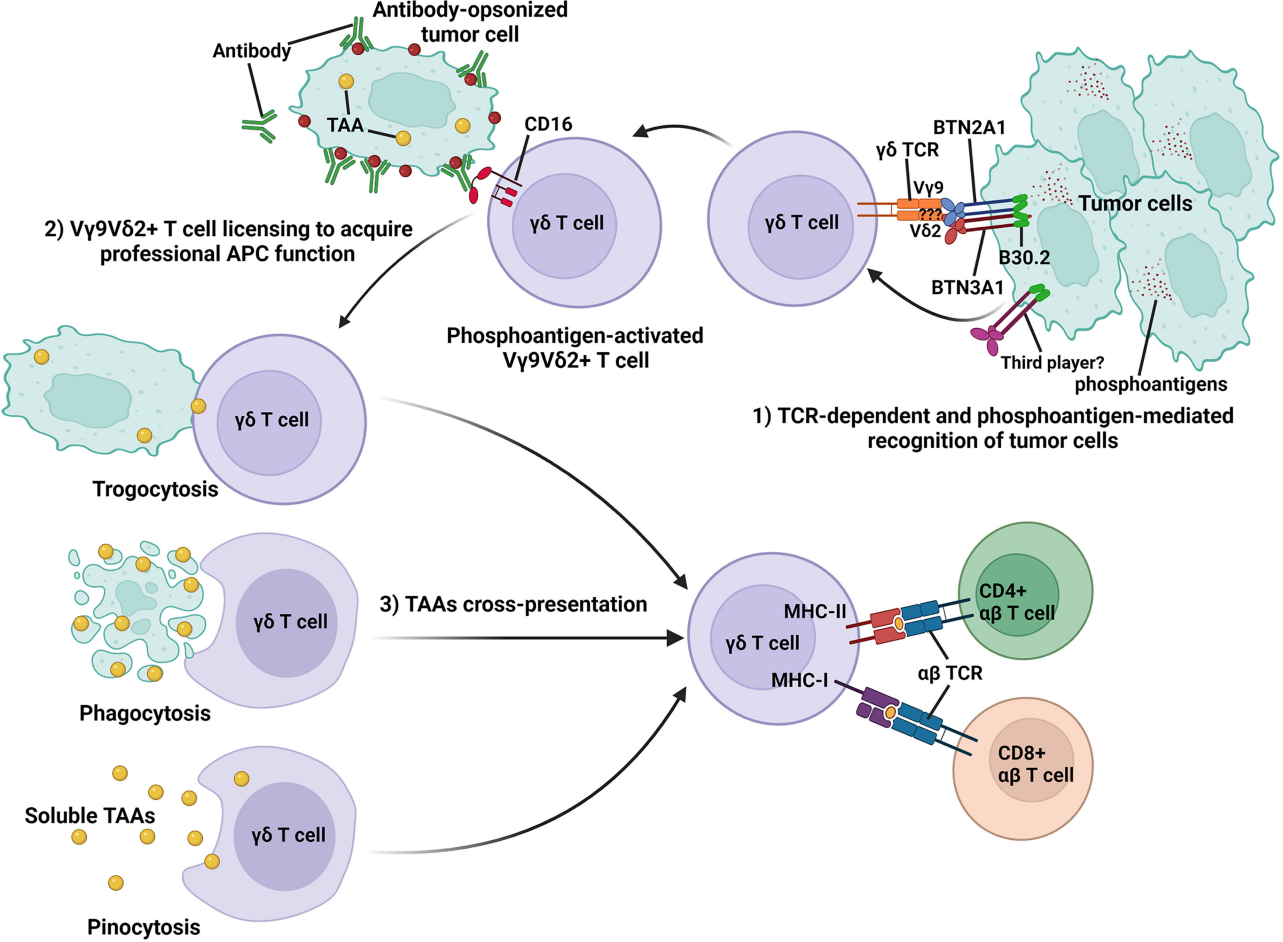
Schematic representation of TCR-dependent and phosphoantigen-mediated recognition of tumor cells by Vγ9Vδ2^+^ T cells and the acquisition of professional APC function by activated Vγ9Vδ2^+^ T cells to cross-present TAAs to antigen-specific CD4^+^ and CD8^+^ αβ T cells. During the Vγ9Vδ2^+^ T-cell activation process, accumulated phosphoantigens in tumor cells bind to the intracellular B30.2 domain of BTN3A1. Following phosphoantigen binding, BTN3A1 undergoes conformational changes and induces the interaction between the intracellular domains of BTN2A1 and BTN3A1. BTN2A1 directly binds the TCR Vγ9 chain and leads to T-cell activation in concert with at least one additional ligand. Activated Vγ9Vδ2^+^ T cells can recognize antibody-opsonized tumor cell *via* CD16 (FcγRIII) and are licensed to acquire professional APC function *via* trogocytosis, phagocytosis, and pinocytosis and cross-present antigens from tumor cells to antigen-specific CD4^+^ and CD8^+^ αβ T cells.

Vγ9Vδ2^+^ T cells are the most abundant Vδ cell population found in peripheral blood and are activated by phosphorylated non-protein metabolites called phosphoantigens *via* the BTN2A1/BTN3A1 complexes in a TCR-dependent manner (3, 11, 23, 24). Phosphoantigens are derived from the mevalonate pathway as an intermediate metabolite known as isopentenyl pyrophosphate (IPP) (25), or are generated in the microbial non-mevalonate isoprenoid synthesis pathway as (E)-4-hydroxy-3-methyl-but-2-enyl-pyrophosphate (HMBPP) (26). Following phosphoantigen binding to the intracellular B30.2 domains of BTN3A1 in tumor or pathogen-infected cells (27), BTN3A1 undergoes a conformational change (28–30) and promotes the interaction between BTN2A1 and BTN3A1 intracellular domains (31). Subsequently, the germline-encoded regions of the TCR Vγ9 chain directly bind to BTN2A1 on tumor cells (3, 32, 33), as described by us and confirmed later by others (34–36). An additional but yet to be identified ligand is likely to bind to a separate region within the complementarity-determining region 2δ (CDR2δ) and CDR3γ of the Vγ9Vδ2 TCR for phosphoantigen-mediated Vγ9Vδ2^+^ T-cell activation (3, 33). In concert with BTN2A1, the phosphoantigen-induced conformational change of BTN3A1 then leads to Vγ9Vδ2^+^ T-cell activation (31, 33–36) (**Figure 1**). Accordingly, dysregulation of the mevalonate pathway in tumors was shown to cause activation of Vγ9Vδ2^+^ T cells *via* IPP accumulation (37) and induced γδ T-cell chemotaxis toward tumor cells (38, 39). Activated Vγ9Vδ2^+^ T cells are capable of inducing cytotoxicity *via* secretion of Th1 cytokines such as tumor necrosis factor-α (TNF-α) and interferon-γ (IFN-γ), pro-apoptotic protease granzyme B, and cytolytic granules containing pore-forming perforin molecules (40–44). Therefore, many clinical studies used aminobisphosphonates (e.g., zoledronate and pamidronate) to inhibit farnesyl pyrophosphate synthase in the mevalonate pathway to promote accumulation of IPP in cells, or synthetic phosphoantigen analogues such as bromohydrin pyrophosphate (BrHPP) and 2-methyl-3-butenyl-1-pyrophosphate (2M3B1PP), to activate Vγ9Vδ2^+^ T cells in cancer patients (19, 45–47). In recent years, however, agonist antibodies against BTN3A such as clone 20.1 (48–51), CTX-2026 (52), and ICT-01 (53) have been explored as a phosphoantigen-independent approach to activate Vγ9Vδ2^+^ T cells for targeted cell killing. Moreover, Vγ9Vδ2^+^ T cells can be activated by other ligands including human MutS homolog 2, stress-induced MHC class I chain-related antigens A and B (MICA/MICB), UL16-binding proteins (ULBPs), nectin-like-5, staphylococcal enterotoxins (SEs), toxic shock syndrome toxin 1 (TSST-1), and F1-ATPase-apolipoprotein-AI through surface receptors, natural killer group 2D (NKG2D), and DNAX accessory molecule-1 (DNAM-1) (12, 13, 17, 19, 54, 55). Other than direct targeted cell killing, activated Vγ9Vδ2^+^ T cells have been implicated to directly or indirectly interact with a range of immune cells: αβ T cells (56–63), B cells (64–72), natural killer (NK) cells (73–75), monocytes (76–78), macrophages (79–82), neutrophils (78, 83–86), monocyte-derived dendritic cells (moDCs) (87–93), and DCs (72, 76, 94–96), and influence the outcome of the immune responses. The underlying mechanisms of such γδ T-cell crosstalk with other immune cells are summarized in **Table 1** and will be thoroughly discussed in the following sections.

**Table 1:** Summary of distinct γδ T-cell subset interactions with other immune cells.

γδ T-cell subset	Crosstalk target	Comments	References
Pan-γδ	CD4^+^ and CD8^+^ αβ T cells	Activated γδ T cells were capable of professional phagocytosis to mediate presentation of antigens to CD4^+^ and CD8^+^ αβ T cells	(62, 97, 98)
	CD4^+^ and CD8^+^ αβ T cells; CD4^+^ CD25^+^ Treg cells	Tumor-activated γδ T cells induced proliferation and differentiation of CD4^+^ and CD8^+^ αβ T cells, mediated cytotoxic function of CD8^+^ αβ T cells and inhibited immunosuppression effect by CD4^+^ CD25^+^ Treg cells on CD4^+^ CD25^-^ αβ T cells	(99)
	B cells	Phosphoantigen-activated γδ T cells provided B-cell help for the downstream production of IgA, IgG, and IgM antibodies	(68)
	NK cells	IPP-activated γδ T cells upregulated CD137L expression and co-stimulated CD25^hi^, CD54^hi^, CD69^hi^, CD137^hi^ NK cells *via* CD137/CD137L (4-1BB/4-1BBL) interactions to promote NK cell-mediated cytotoxicity against tumors	(73, 75)
	NK cells	IPP-activated γδ T cells expressed ICOS and co-stimulated NK cell activation through ICOS/ICOS-L interactions, leading to increased CD137/CD137L signaling and acquisition of NK cell-mediated DC editing function	(100, 101)
Vδ1^+^	CD4^+^ and CD8^+^ αβ T cells; DCs	Activated Vδ1^+^ γδ T cells suppressed proliferation and IL-2 production by both CD4^+^ and CD8^+^ αβ T cells and impaired the maturation and function of DCs. The suppressive activity of activated Vδ1^+^ γδ T cells was mediated by TLR8 signaling pathway	(102)
	DCs	Tumor-derived CXCL10 increased the expansion of Vδ1^+^ γδ Treg cells that infiltrated solid tumors and either induced immune-senescence in DCs or killed DCs	(102–107)
Vδ2^+^	CD4^+^ αβ T cells	IPP-activated Vγ9Vδ2^+^ T cells acquired professional APC functions by upregulating expression of co-stimulatory (CD40, CD80, and CD86), MHC class II and lymph node-homing CCR7 receptors, presented exogenous antigen and induced naïve autologous CD4^+^ αβ T cells to proliferate and differentiate into T helper, Th1 subset	(56)
	CD8^+^ αβ T cells	IPP-activated HLA-A2^+^ Vγ9Vδ2^+^ T cells could uptake soluble antigens, processed and cross-presented immunodominant or subdominant HLA-A2-restricted peptides and primed naïve CD8^+^ αβ T cells for proliferation and effector cell function	(57–61)
	CD8^+^ αβ T cells	IPP-activated Vγ9Vδ2^+^ T cells upregulated CD36 expression to mediate apoptotic and live tumor cells uptake, cross-presentation, and induction of TAA-specific CD8^+^ αβ T-cell response	(108)
	B cells	Vγ9Vδ2^+^ T cells promoted the development of antibody-producing B cells *via* immunoglobulin class switching	(65–67, 69)
	B cells	Activated Vγ9Vδ2^+^ T cells with functional CCR7 expression induced transient lymph node-homing and clustering within B-cell zones of germinal centers in lymphoid tissues	(64, 68)
	NK cells	IPP-activated Vδ2^+^ γδ T cells induced cytotoxicity against CD56^+^ DC-like cells and prematurely terminated NK cell response	(74)
	Monocytes	IPP- or HMBPP-activated Vδ2^+^ γδ T cells induced downregulation of CD14, and upregulation of CD40, CD86, and HLA-DR on monocytes	(76, 77)
	Macrophages	Macrophages recruited Vδ2^+^ γδ T cells to the site of infection *via* IP-10 and CXCR3; once there they were able to drive the local cytotoxic response *via* granzyme and perforin release or Fas ligand binding	(79–82)
	Neutrophils	IPP- or HMBPP-activated Vγ9Vδ2^+^ T cells can induce neutrophil recruitment, migration, adhesion, activation, phagocytosis, and degranulation	(78, 83, 86)
	Neutrophils	TNF-α secretion by γδ T cells induces reactive oxygen species, arginase-1, and serine protease production from neutrophils, which subsequently inhibits CD25 and CD69 expression, IFN-γ production, and cell proliferation of Vδ2^+^ γδ T cells	(84–86)
	DCs	Activated Vγ9Vδ2^+^ T cells secreted IFN-γ and TNF-α and promoted maturation of antigen-expressing immature moDCs in circulation	(87–91, 93)
Vδ3^+^	DCs	Activated Vδ3^+^ γδ T cells induced immature moDCs to upregulate APC markers CD40, CD83, CD86, and HLA-DR and secreted IL-10 and IL-12. Vδ3^+^ γδ T cell-mediated moDC maturation involved CD1d recognition but not CD40/CD40L interaction. Vδ3^+^ γδ T cell-matured moDCs induced activation of naïve allogeneic T cells.	(109)

The non-Vδ2 γδ T cells are mostly identified with Vδ1^+^ or Vδ3^+^ TCR chain usage and are localized in the skin, large intestine, spleen, and liver (6, 12, 54). Several studies have shown that Vδ1^+^ γδ T cells recognize CD1c-phosphomycoketide (110), CD1d-α-GalCer (111), CD1d-sulfatide (112, 113), R-phycoerythrin (PE) (114), ephrin receptor A2 (EphA2) (115), and MHC-related protein 1 (MR1) (116) ligands, and play a crucial role for anti-tumor responses (117–124). Similar to Vδ2^+^ γδ T cells, the NKG2D-expressing Vδ1^+^ γδ T cells can be activated by stress-inducible MICA/MICB and ULBP1–6 family proteins, which are frequently upregulated in tumor cells (8, 11). Ligand-bound NKG2D induces cytolytic functions of γδ T cells *via* granzyme B and perforin secretion to mediate tumor cell killing (125). Several studies have utilized Vδ1^+^ γδ T-cell populations for adoptive cancer immunotherapy (8, 10, 126), but the clinical outcome so far was limited. The less frequent Vδ3^+^ γδ T cells were shown to recognize and kill CD1d^+^ target cells (109) and are activated by annexin A2 ligands on tumor cells that are upregulated under oxidative stress conditions (127). Interestingly, the binding affinity of the Vδ1^+^ and Vδ3^+^ γδ TCR ligands identified thus far falls within the range of 3 to 150 µM (55, 128), comparable to the well-studied αβ TCR binding affinities for the peptide–MHC complex (129, 130), suggesting a possible shared TCR docking footprint on the bound ligand (131). With the increasing numbers of non-Vδ2 γδ T-cell ligands uncovered so far (8, 55, 116, 132), different strategies have been developed to utilize activated non-Vδ2 γδ T cells for cancer immunotherapy (10, 19, 128). Of note, activated non-Vδ2 γδ T cells have also been implicated to modulate other immune cells (**Table 1**) including αβ T cells (102), B cells (133–135), DCs (89, 102–107, 109, 136, 137), macrophages (70, 138), and neutrophils (139).

Human Vδ2^+^ γδ T cells represent ~0.5% to 10% of all circulating T lymphocytes in healthy adults and can undergo rapid expansion of up to 60% in the periphery during infections, and form between 20% to 30% of total infiltrating CD3^+^ T cells in the early stage of disease onset (11, 17). Activated Vδ1^+^ and Vδ2^+^ γδ T cells upregulate various C-C chemokine receptor (CCR) such as CCR1 and CCR8 (140), CCR2 (141), CCR5 (142), and C-X-C chemokine receptor 3 (CXCR3) (107) to mediate infiltration into the tumor microenvironment (TME). Additionally, tumor cells and tumor-derived fibroblasts express chemokine ligand 2 (CCL2) (141), IFN-γ-inducible protein 10 (IP-10) (107), monocyte chemoattractant protein 1 (MCP-1), macrophage inflammatory protein 1α (MIP-1α), MIP-1β, and regulated on activation, normal T cell expressed and secreted (RANTES) to promote recruitment of activated Vδ1^+^ and Vδ2^+^ γδ T cells to the TME (140). Once recruited into the TME, tumor-infiltrating Vδ1^+^ and Vδ2^+^ γδ T cells can eliminate tumor cells *via* TNF-related apoptosis-inducing ligand (TRAIL) (143), Fas/Fas ligand pathway (144), induction of antibody-dependent cellular cytotoxicity (ADCC) on antibody-opsonized tumor cells through CD16 (FcγRIII) (60, 145, 146), perforin/granzymes, IFN-γ/TNF-α secretion, and NKG2D-mediated cytotoxicity (13, 147). As a result of the complex interplay between TME and tumor-infiltrating γδ T cells, activated γδ T cells can be functionally polarized to become the anti-tumor Th1 and follicular Th (Tfh) cells or the pro-tumor Th17 and T regulatory (Treg) cells (12, 132). For example, IPP-activated Vγ9Vδ2^+^ T cells can be polarized into three distinct subsets based on the presence of different cytokines in the microenvironment: Th1 [interleukin-12 (IL-12) and anti-IL-4 antibody] (148), Th2 (IL-4 and anti-IL-12 antibody) (148), and Th17 [IL-1β, transforming growth factor β (TGF-β), IL-6 and IL-23] (149). Recent reviews on the topic of γδ T-cell polarization has provided comprehensive insight into the different role of γδ Th1, Th2, Th17, Tfh, and Treg cells, and we refer readers to these excellent publications (7, 8, 11, 54, 150–153).

Importantly, the presence of tumor-infiltrating γδ T cells was shown to be the most favorable prognostic marker for overall cancer patients survival in 25 different cancer types and solid tumors (non-brain tumor) (4). Their role in cancer immunosurveillance was clearly evidenced and validated in many tumor models and clinical studies including cutaneous carcinoma (154), melanoma (119, 155, 156), lymphoma (157–159), leukemia (44, 117, 160, 161), gastric (162), colorectal (43, 163, 164), kidney (41), prostate (165, 166), and pancreatic (143) cancers. The ability of γδ T cells to produce large quantities of cytokines and chemokines rapidly and their tendency to reside in blood circulation or in non-lymphoid tissues (e.g., skin, intestines, and lungs) (8, 16, 17), helps to provide the first line of immunosurveillance against aberrant cell growth and infectious diseases, and bridges the innate and adaptive immune responses. Thus, it is important to understand the crosstalk between γδ T cells and other immune cells in the TME and to harness this knowledge for effective cancer immunotherapy development.

## Crosstalk between γδ T cells and αβ T cells

The role of antigen processing and presentation to αβ T cells is mostly associated with the classical professional antigen-presenting cells (APCs) like DCs, macrophages, and B cells (167, 168). However, with the unexpected discovery by Brandes et al., it was shown that activated but not resting human Vγ9Vδ2^+^ T cells were also capable of acquiring professional APC functions (56). Indeed, activated Vγ9Vδ2^+^ T cells isolated from both healthy individuals and cancer patients’ peripheral blood mononuclear cell (PBMC) exhibited potent APC functions to stimulate robust antigen-specific αβ T-cell responses (169).

During the activation process, human Vγ9Vδ2^+^ T cells can rapidly gain APC functions by upregulating co-stimulatory (CD40, CD80, and CD86), MHC class I and II molecules (56, 57, 61, 62, 97, 108, 169), and transiently expressed lymph node-homing markers, chemokine receptor CCR4 and CCR7 (62, 68, 97). This allows recruitment of activated γδ T cells from the peripheral sites to secondary lymphoid tissues for antigen presentation and bridges the early phase of rapid innate-like γδ T-cell response to microbial or tumor antigens with the later phase of adaptive immune response involving the antigen-specific CD4^+^ and CD8^+^ αβ T cells (14, 15, 17, 168, 170). In a study by Himoudi et al., it was shown that activated human Vγ9Vδ2^+^ T cells were “licensed” to acquire their APC functions through recognition of antibody-opsonized tumor cells, mediated targeted cell killing by their innate cytotoxicity, and subsequently helped to release tumor-associated antigens (TAAs) into the surrounding microenvironment (60). These TAAs can be taken up by activated γδ T cells *via* phagocytosis (62, 97, 98, 108), trogocytosis (171), or pinocytosis (57, 58), processed and presented on the cell surface for priming and induction of naïve αβ T cells (59, 60) (**Figure 1**). Furthermore, it was shown that Vγ9Vδ2^+^ γδ T cells can uptake microbes and soluble antigens *via* CD16-mediated phagocytosis, a process that can lead to functional antigen processing and presentation on MHC class II (98), and cross-presentation of immunodominant MHC class I peptides to antigen-specific CD8^+^ αβ T cells (58, 60, 61). This notion was further supported by the identification of Vγ9Vδ2^+^ T cells in malaria patients that readily acquired APC functions upon infection and induced CD4^+^ and CD8^+^ αβ T-cell activation (61). Interestingly, it was also demonstrated that activated Vγ9Vδ2^+^ T cells can uptake CD1d-containing membrane fragments from phosphoantigen expressing Cd1d^+^ target cells *via* trogocytosis, leading to the presentation of CD1d-restricted antigen and the activation of Vα24Vβ11^+^ invariant natural killer T cells (iNKT) (172).

**Figure 2:**
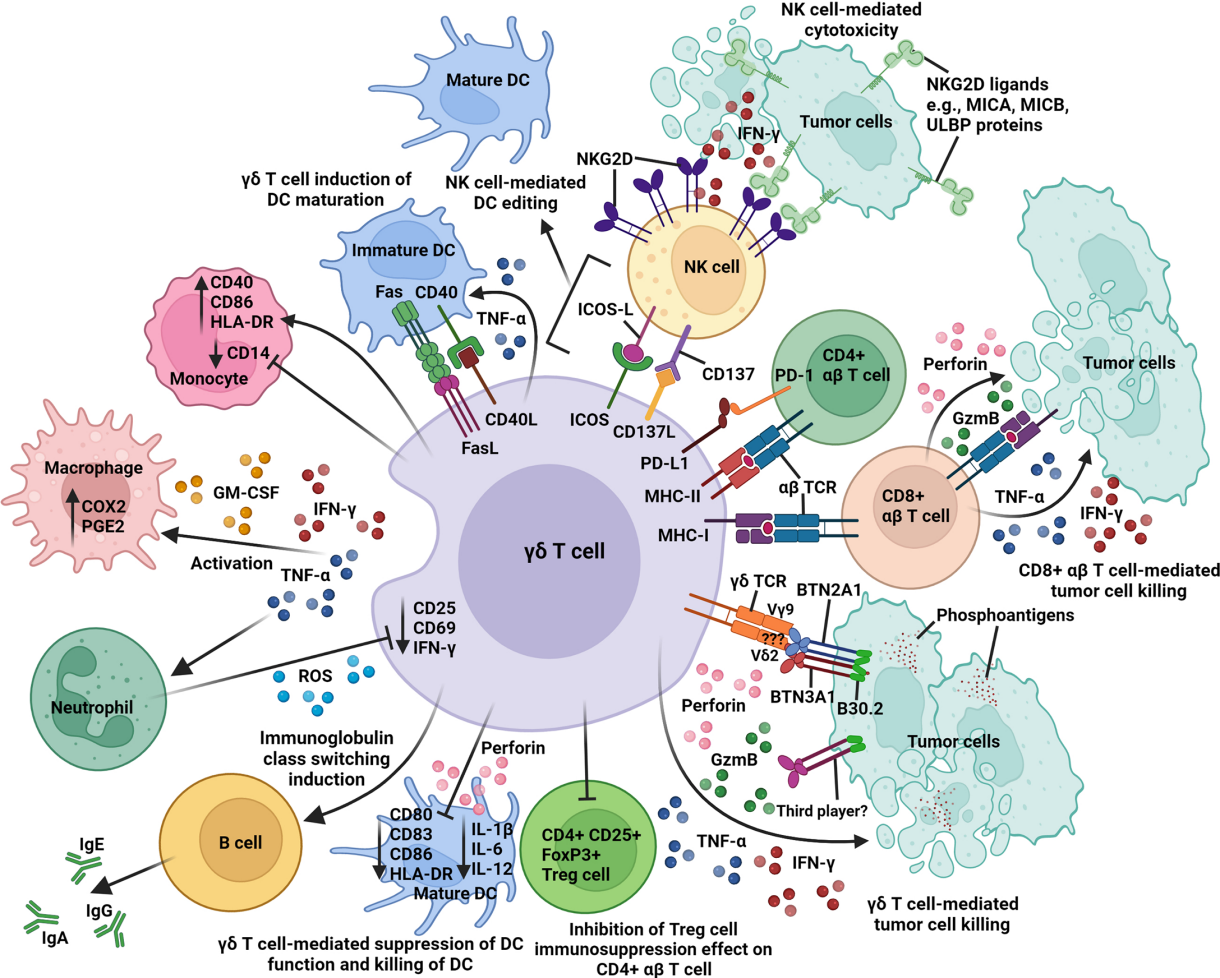
An overview of the intricate network of immune interactions between γδ T cell and other immune cells in the tumor microenvironment. Activated γδ T cells express different surface receptors and molecules (γδ TCR, ICOS, MHC class I and II), ligands (CD40L, CD137L, FasL, and PD-L1), cytokines (IFN-γ and TNF-α), and GM-CSF for contact-dependent and independent crosstalk with tumor cells, CD4^+^ and CD8^+^ αβ T cells, NK cells, DCs, macrophages, and neutrophils. Activated γδ T cells cross-present antigens to CD4^+^ and CD8^+^ αβ T cells; induce B-cell immunoglobulin class switching; co-stimulate NK cells *via* CD137/CD137L and ICOS/ICOS-L interactions; induce upregulation of CD40, CD86, and HLA-DR expression on monocyte; promote DC maturation *via* CD40/CD40L and Fas/FasL interactions; and inhibit the immunosuppression function of CD4^+^ CD25^+^ FoxP3^+^ Treg cells on CD4^+^ αβ T-cell activity. In contrast, activated γδ T cells can also suppress DC function (downregulation of CD80, CD83, CD86, HLA-DR, IL-1β, IL-6, and IL-12) and mediate DC killing *via* perforin release. Butyrophilin 2A1 and 3A1 (BTN2A1 and BTN3A1); cyclooxygenase-2 (COX2); granulocyte-macrophage colony stimulating factor (GM-CSF); granzyme B (GzmB); human leukocyte antigen-DR (HLA-DR); immunoglobulin A, E, or G (IgA, IgE, or IgG); inducible T-cell co-stimulator (ICOS); ICOS ligand (ICOS-L); interferon-γ (IFN-γ); major histocompatibility complex class I and II (MHC-I and -II); MHC class I chain-related antigens A and B (MICA and MICB); natural killer group 2D (NKG2D); programmed cell death 1 (PD-1); PD-1 ligand 1 (PD-L1); prostaglandin E2 (PGE2); reactive oxygen species (ROS); T-cell receptor (TCR); tumor necrosis factor-α (TNF-α); UL16-binding protein (ULBP).

When compared to activated αβ T cells and monocytes, activated Vγ9Vδ2^+^ T cells were shown to be more efficient in presenting antigens and induced 100-fold higher proliferative responses in naïve CD4^+^ αβ T cells (56). Activated Vγ9Vδ2^+^ T cells were also able to cross-present antigens to CD8^+^ αβ T cells with a higher efficiency and reproducibility (57), and induced less CD4^+^ CD25^hi^ FoxP3^+^ Treg cell expansion than moDCs (59). Similar results were seen under pathological condition, when it was shown that γδ T cells isolated from gastric cancer patients can acquire APC functions upon activation with cells derived from autologous tumor tissues (99). These clinically relevant tumor-activated γδ T cells induced strong antigen-specific CD4^+^ and CD8^+^ αβ T-cell responses and prevented immunosuppression mediated by CD4^+^ CD25^+^ Treg cells (99) (**Figure 2**). Of note, Muto et al. showed that resting Vγ9Vδ2^+^ T cells can significantly upregulate the expression of scavenger receptor CD36 during activation and that this was mediated by a key transcription factor, CCAAT/enhancer-binding protein α (C/EBPα), that supports acquisition of APC functions in activated Vγ9Vδ2^+^ T cells (108). In contrast, resting αβ T cells expressed a low level of CD36 and did not upregulate it upon activation (108). In DCs and macrophages, the CD36 receptor was shown to facilitate the uptake of apoptotic cells and cross-presentation (173, 174), potentially explaining the induction of a stronger antigen-specific αβ T-cell response by activated Vγ9Vδ2^+^ T-cell APC.

The ability to migrate to the tumor site and cross-present TAAs to αβ T cells was also retained when Vδ1^+^ and Vδ2^+^ γδ T cells were engineered to express tumor-specific chimeric antigen receptors (CARs) and resulted in an increased cytotoxic level against tumor cells (175). Hence, activated Vγ9Vδ2^+^ T cells can process and present antigens and provide critical co-stimulatory signals to prime and induce naïve CD4^+^ (56) and CD8^+^ (57) αβ T cells for proliferation, differentiation, and cytokine production and to mediate cytotoxic responses against tumors and pathogen-infected cells (176–179). This remarkable ability of γδ T cells to uptake and present antigens and prime αβ T cells has been highlighted by Vantourout et al. (168), and the accumulated data so far have illustrated the potential of harnessing the APC functions of γδ T cells to crosstalk with αβ T cells for immunotherapy development.

Given their natural tropism for TME (14, 119, 175, 180–182), activated γδ T cells could hence be utilized to prolong the intratumoral immune response by cross-presenting TAAs to other tumor-infiltrating lymphocytes and provide an early source of IFN-γ to expand and increase immunogenicity of TAA-specific αβ T cells within the TME (155, 183, 184), and to upregulate expression of MHC class I and II on tumor cells (185, 186) for αβ T cell-mediated killing (**Figure 2**). The presence of tumor-infiltrating γδ T cells within the TME as revealed by genomic data analysis in over 18,000 human tumors has uncovered a strong correlation to good prognosis (4). In the context of cancer immunotherapy, the capability of activated γδ T cells to cross-present TAAs to αβ T cells could be further boosted through the “licensing” pathway (60, 187) by using therapeutic monoclonal antibodies against tumor cells, e.g., rituximab (anti-CD20) and trastuzumab (anti-HER2/neu) (145, 188, 189). Such combination treatment could greatly improve the outcome of γδ T-cell cancer immunotherapy.

Activated Vγ9Vδ2^+^ T cells can also modulate αβ T-cell activity indirectly by co-stimulating NK cells *via* inducible T-cell co-stimulator (ICOS)/ICOS-L and CD137/CD137L engagements to enhance IFN-γ and TNF-α production (100, 101), which, in turn, helps to support αβ T-cell activation (190). Another study has shown that activated Vγ9Vδ2^+^ T cells can induce B-cell and DC maturation and subsequently leads to alloreactive stimulation of αβ T-cell proliferation and IFN-γ production by mature B cells and DCs (72). The interactions between γδ T cells and other immune cells (B cells, DCs, and NK cells) will be discussed later in this review.

Despite their ability to exert positive immune modulation functions on αβ T cells, activated γδ T cells can also negatively regulate αβ T-cell response by upregulating an immune checkpoint inhibitory ligand, programmed cell death 1 ligand 1 (PD-L1) (11, 151, 191). The suppressive phenotype of activated Vδ2^+^ γδ T cells on autologous αβ T cells was shown to be mediated by the PD-1/PD-L1 interactions and correlated well with the strength of Vδ2^+^ γδ TCR signaling during the activation process but was independent of TGF-β and FoxP3 expression (192) (**Figure 2**). Daley et al. showed that tumor-infiltrating γδ T cells with high expression levels of checkpoint inhibitory ligands PD-L1 and Galectin-9 could inhibit αβ T-cell activation through checkpoint receptor ligation (193). The immunosuppressive effect can also be mediated by the interaction between CD86 on activated Vδ2^+^ γδ T cell and cytotoxic T lymphocyte-associated antigen 4 (CTLA-4) on activated αβ T cells (191). Such γδ T cell-mediated immunosuppression of αβ T cells, however, can be significantly reduced by disrupting PD-1/PD-L1 and CTLA-4/CD86 interactions with blocking antibodies (191, 192). Furthermore, Peng et al. identified tumor-infiltrating Vδ1^+^ γδ T cells that could suppress naïve/effector αβ T-cell proliferation and IL-2 production through the Toll-like receptor (TLR) 8 signaling pathway and may lead to tumor immune escape (102). The immunosuppressive activity of Vδ1^+^ γδ T cells can be reversed using TLR8 ligands, and this signaling involved the myeloid differentiation primary response 88 (MyD88), TNFR-associated factor 6 (TRAF6), IKB kinase α (IKKα), IKKβ, and mitogen-activated protein kinase 14 (MAPK14), but not transforming growth factor-β-activated kinase 1 (TAK1), Jun N-terminal kinase (JNK), and extracellular signal-regulated kinase (ERK) molecules in Vδ1^+^ γδ T cells (102). It was also reported that γδ^+^ NKG2A^+^ intraepithelial lymphocytes (IELs) can mediate suppression of CD8^+^ αβ^+^ IEL cytotoxic responses (IFN-γ and granzyme B) in patients with celiac disease through TGF-β secretion (194). The immunosuppressive effect on CD8^+^ αβ^+^ IELs can be further enhanced upon γδ^+^ IELs NKG2A receptor ligation with the cognate ligand, human leukocyte antigen-E (HLA-E) (194). This immunosuppressive effect can be reduced by blocking NKG2A/HLA-E interaction and TGF-β with blocking antibodies (194). Therefore, it is important to consider these negative immunomodulatory roles of γδ T cells when designing novel immunotherapeutics.

Apart from the PD-1/PD-L1 and CTLA-4/CD86 immune checkpoint axes, other non-conventional checkpoint receptors [killer Ig-like inhibitory receptors (KIRs), Ig-like transcript 2 (ILT-2), and NKG2A] can be expressed on Vγ9Vδ2^+^ T cells, inhibit their cytotoxic function, and prevent tumor cell lysis upon recognition of specific HLA class I ligands on tumor cells (195–203). In this context, the presentation of HLA class I molecules on tumor cells can be a double-edged sword. On one hand, it facilitates the presentation of antigenic peptides to activate CD8^+^ αβ T cells, but at the same time, it can also inhibit the activation of Vγ9Vδ2^+^ T cells. Such inhibitory signals on immune cells mediated by KIRs, ILT-2, or NKG2A can be blocked using monoclonal antibodies targeting KIRs (lirilumab and IPH4102), ILT-2 (anti-ILT-2, anti-HLA-G1, anti-FasL), or NKG2A (monalizumab) (204, 205). In a study by André et al., treatment with monalizumab indeed led to enhanced anti-tumor immune responses elicited by T and NK cells (206). As a type 2 inhibitory membrane receptor, NKG2A carries cytoplasmic immunoreceptor tyrosine-based inhibitory motifs (ITIMs) and forms heterodimers with CD94 to recognize non-classical HLA-E molecule (207). Many human tumors have been shown to express HLA-E including in the colon, cervical, endometrial, head and neck, liver, lung, pancreas, ovarian, and stomach (206). Moreover, a majority of Vγ9Vδ2^+^ T cells in healthy individuals express NKG2A/CD94 (197, 198, 200, 208), and the expression levels can be induced by IL-15 and TGF-β (209, 210). Therefore, treatments targeting these non-conventional checkpoint receptors on Vγ9Vδ2^+^ T cells (KIRs, ILT-2, and NKG2A) to disrupt the interactions with their respective HLA class I ligands on tumor cells (HLA-C, HLA-G, and HLA-E) may help to enhance the effectiveness of Vγ9Vδ2^+^ T cell-based tumor immunotherapy.

Recent work by Payne et al. suggests that BTN3A, itself part of the molecular complex required for phosphoantigen-mediated activation of Vγ9Vδ2^+^ T cells, can also inhibit tumor-reactive CD8^+^ αβ T cells when bound to N-mannosylated residues of CD45 by preventing its segregation from the immunological synapse (52). In this study, the suppression of αβ T-cell activation was shown to involve BTN3A1 but not BTN2A1, and the immunosuppressive effect could be blocked by BTN3A1-specific monoclonal antibodies such as clone 20.1, 103.2, and CTX-2026 (52). Targeting BTN3A1 with the agonistic antibody CTX-2026 induced BTN3A1 switching from immunosuppressive to immunostimulatory conformations and promoted coordinated Vγ9Vδ2^+^ and CD8^+^ αβ T-cell anti-tumor responses against BTN3A1^+^ tumors (52). Hence, BTN3A1 may be an attractive immune target for intervention to orchestrate effective and coordinated γδ and αβ T-cell anti-tumor responses.

## Crosstalk between γδ T cells and B cells

γδ T cells have been previously reported to interact with B cells and modulate their immune functions (5, 8, 168, 211, 212). Vγ9Vδ2^+^ T cells can adopt a role similar to T follicular helper (Tfh) cells and provide B-cell help, thereby regulating B-cell maturation. Specifically, a subset of CXCR5^+^ Vγ9Vδ2^+^ T cells present in circulation and in tonsil tissue expresses co-stimulatory molecules (ICOS and CD40L) upon antigen stimulation and secrete cytokines (IL-2, IL-4, and IL-10), which can promote the development of antibody-producing B cells *via* immunoglobulin class switching [including immunoglobulin A (IgA), IgE, IgG1, IgG2, IgG3, and IgG4] (8, 213, 214) in the extra-follicular or within germinal centers (65–67, 69) (**Figure 2**). Furthermore, upon stimulation with IL-21 and HMBPP, activated tonsillar Vγ9Vδ2^+^ T cells can express CXCL13 receptor, CXCR5, induce lymphoid-homing phenotype and clustering in germinal centers, and sustain the production of germinal centers (70, 71). Similarly, IPP-stimulated Vδ2^+^ γδ T cells with functional CCR7 expression can also induce transient lymph node-homing, migration, and clustering of Vδ2^+^ γδ T cells within B-cell zones of germinal centers in lymphoid tissues (64, 68).

Phosphoantigen-activated Vδ2^+^ γδ T cells can additionally induce the expression of B-cell co-stimulatory molecules (CD40L, OX40, CD70, and ICOS) and affect the downstream production of circulating IgA, IgG, and IgM antibodies by B cells (68). In patients with specific mutations (*RAG1* and *CD3D*) that impair αβ T-cell function, γδ T cells are responsible for hyper-IgE syndromes or the elevated production of circulating IgA, IgG, and IgM (215, 216). γδ T cells can also suppress antibody responses *via* the induction of CD4^+^ Foxp3^+^ Treg cells (217). Conversely, some B cells can express BTN2A1 and BTN3A1, required for Vγ9Vδ2^+^ T-cell activation (33–35), thereby directly influencing Vδ2^+^ γδ T-cell activation (218, 219) as shown by early studies using Daudi cells, a B-cell malignancy cell line (Burkitt’s lymphoma) (220–226). Vγ9Vδ2^+^ T cells can directly engage BTN2A1 expressed on B cells *via* the TCR Vγ9 chain (3, 32–36, 227), and in concert with BTN3A1, this results in Vγ9Vδ2^+^ T-cell activation and expansion (101, 212). Hebbeler et al. showed that the Vγ9Vδ2^+^ T cells activated and expanded by phosphoantigen or Daudi B lymphoma cells use public TCR Vγ9 clonotypes, and elicit comparable cytotoxic responses against tumor cells (228). Further investigations revealed that the germline-encoded region between TCR Vγ9 CDR2 and CDR3 is responsible for contacting BTN2A1 on target cells (33, 34). Such findings indicate the inherent property of TCR Vγ9 to recognize diverse range of cell types that express BTN2A1 including B cells (212, 227–229). In addition to BTN2A1 and BTN3A1, B cells also express other closely related BTN molecules such as BTN3A2 (in naïve or germinal center B cells), BTN3A3 (in memory B cells), BTN1A1, and BTN2A2 (3, 50). The contribution of these other BTN molecules in B cells for γδ T-cell activation remains elusive. Similarly, circulating activated B7^+^ CD39^+^ B cells can stimulate Vδ1^+^ γδ T-cell proliferation (133, 134). The Vδ1^+^ γδ T-cell stimulatory ligand is upregulated in B cells upon activation and can induce polyclonal Vδ1^+^ γδ T-cell responses (133). This B cell-mediated immunostimulatory effect on Vδ1^+^ γδ T cells can be blocked with antibodies against B7 and CD39 (133, 212).

In summary, Vγ9Vδ2^+^ T cells can regulate B-cell maturation during development or initiation of an immune response, sustain the production of germinal centers within secondary and possibly tertiary lymphoid structures, and affect the production of circulating (auto)antibodies for humoral immunity (168, 211, 212), while B cells can activate Vδ1^+^ and Vγ9Vδ2^+^ T cells (230).

## Crosstalk between γδ T cells and NK cells

Human NK cells are important innate immune subset for controlling early tumor growth and metastasis through cell-mediated cytotoxicity and show broad reactivity to tumors that escaped immunosurveillance by loss or aberrant MHC class I expression (14, 231, 232). Being a specialized group of innate lymphoid cells (ILCs), NK cell functions are closely regulated by a range of cytokines such as IFN-γ, TNF-α, IL-2, IL-12, IL-15, IL-18, and IL-21 (233, 234). These effector molecules are important for the initiation of anti-viral and anti-tumor immune responses (235–238). However, more established tumors can evade NK cell surveillance by developing resistance to NK cell-mediated cytotoxicity, leading to tumor immune escape (239).

In order to overcome NK-resistant tumors, Maniar et al. showed that activated human NK cells (CD25^hi^, CD54^hi^, CD69^hi^, and CD137^hi^) increased surface expression of natural NKG2D receptors to promote tumor cytolysis and death (73). NKG2D is a lectin-like type 2 transmembrane receptor mostly expressed by human NK cells and binds to MHC-related ligands such as ULBPs, MICA, and MICB, which are highly expressed in tumor cells but rarely in healthy cells (231, 240). IPP-activated Vδ2^+^ γδ T cells upregulate CD137L (4-1BBL), engage with CD137^+^ NK cells, and can in turn lead to enhanced NKG2D expression and NK cell-mediated cytotoxicity against tumors (73) (**Figure 2**), highlighting a potential key role for γδ T cells in this process. CD137 or 4-1BB is a member of the tumor necrosis factor receptor superfamily (TNFRSF) and is expressed by a range of immune cells (190). Expression of CD137 on NK cells is induced by IL-2 and IL-15, and following CD137 signaling, it promotes NK cell proliferation and production of IFN-γ, which, in turn, can support NK tumor effector functions (101, 190). This finding was further corroborated by Liu et al., and they demonstrated that in the context of liver fibrosis, γδ T cells engaged with conventional and liver-resident NK cells through CD137/CD137L interactions to promote NK cell-mediated cytotoxicity against activated hepatic stellate cells and conferred immune protection (75).

Similar to NK and CD8^+^ αβ T cells, human γδ T cells also express NKG2D to detect stress-inducible ligands on tumors and pathogen-infected cells (125, 241–245). Several studies have shown that NKG2D ligation to its cognate ligand can co-stimulate Vγ9Vδ2^+^ T-cell activation (CD25 and CD69 upregulation) and promotes the release of IFN-γ, TNF-α, and cytolytic granules to mediate killing of NKG2D ligand-expressing tumors (163, 246–251). In the context of leukemia and lymphoma cell recognition by Vγ9Vδ2^+^ T cells, it was reported that tumor-expressed ULBP1 was a strong marker for tumors susceptible to Vγ9Vδ2^+^ T cell-mediated cytotoxicity (252). Similarly, it was shown that ULBP1 overexpression in tumor cells can lead to enhanced killing by Vγ9Vδ2^+^ T cells (253). Hence, blocking NKG2D-mediated Vγ9Vδ2^+^ T-cell recognition of tumor cells with anti-NKG2D and anti-MICA/B monoclonal antibodies inhibits tumor cell killing to varying degrees (247, 249, 253). Vδ1^+^ γδ T cells can also recognize and kill NKG2D ligand-expressing tumors *via* NKG2D receptor (8, 11, 245, 254). The number of Vδ1^+^ γδ T cells and ULBP3 expression level are negatively correlated with disease progression in chronic lymphocytic leukemia patients (254). A study reported by Kamei et al. demonstrated a longer overall survival in gastric cancer patients with high expression levels of NKG2D and ULBP1 (255). Hence, upregulation of stress-inducible NKG2D ligand in tumor cells and NKG2D receptor in tumor-infiltrating immune cells can help to orchestrate concerted NKG2D-mediated NK, CD8^+^ αβ, and γδ T-cell anti-tumor responses within the TME. Of note, several anti-cancer drugs have been found to induce expression of NKG2D ligand in tumor cells, including the proteasome inhibitor bortezomib and the alkylating agent temozolomide, and these can help to promote tumor cell lysis by NK and γδ T cells (256, 257). Therefore, it is feasible to target NKG2D and its ligands for γδ T cell-based immunotherapy development.

It was later shown that IPP-activated Vγ9Vδ2^+^ T cells can upregulate ICOS and signal NK cells *via* ICOS/ICOS-L engagement to promote CD69 and CD137 expression, which then leads to enhanced production of IFN-γ, TNF-α, MIP-1β, I-309, RANTES, and soluble Fas ligand by activated NK cells (100). Such ICOS/ICOS-L-mediated crosstalk enables NK cells to acquire the “license” to kill mature DCs that may play a role in inflammation and tumor growth (100). These studies have uncovered the immunomodulatory role of IPP-activated Vγ9Vδ2^+^ T cells to circumvent NK-resistant tumors and to promote NK-mediated DC editing function by modulating NK cell cytotoxicity through CD137/CD137L and ICOS/ICOS-L engagements (73, 101) (**Figure 2**). Such findings will provide an alternative strategy for γδ T cell-based immunotherapy development against difficult-to-treat solid tumors or to prevent metastasis (239, 258, 259).

However, NK cell activity can also be negatively regulated by γδ T cells. Zoledronate-activated Vδ2^+^ γδ T cells not only can co-stimulate early NK cell activation for IFN-γ production but also lead to premature ending of the response by inducing cytotoxicity against CD56^+^ DC-like cells (74). In the absence of activated Vδ2^+^ γδ T cells, CD56^+^ DC-like cells survived (74) and maintained NK cell activity through secretion of NK cell-activating cytokines such as IL-1β and IL-18 (260, 261). Therefore, further studies will help to provide a better understanding of the immunosuppressive role of Vδ2^+^ γδ T cells on NK cells.

## Crosstalk between γδ T cells and monocytes/macrophages

γδ T cells share many of their innate functions with other immune cell subsets, including NK cells, monocytes, and macrophages (56, 98, 262, 263). These are integral to the innate inflammatory response against infectious pathogens and tumors, which, in turn, activates a strong and targeted adaptive immune response (170). While the hallmark of Vγ9Vδ2^+^ T cell is recognition of phosphoantigens produced by bacteria-infected or tumor cells (25, 264), monocytes are adept at potentiating this process by taking up and accumulating phosphoantigen for subsequent presentation to γδ T cells (262, 263). Conversely, the prototypical roles of myeloid cells, such as phagocytosis and MHC class II presentation, are also shared by Vγ9Vδ2^+^ T cells, which can act as professional APCs (56, 98). The close interconnection between these cell types and partial redundancy in functional properties denotes multiple implications for tumor immunity.

Vγ9Vδ2^+^ T cells have been shown to activate monocytes, induce adhesion and aggregation, and increase their survival (76, 265). This occurs *via* production of inflammatory molecules including IFN-γ, TNF-α, granulocyte-macrophage colony stimulating factor (GM-CSF), lymphocyte function-associated antigen 1 (LFA-1), and CCL2 (76, 78). In turn, this leads to changes in monocyte markers such as downregulation of CD14, and upregulation of CD40, CD86, and HLA-DR (76, 77) (**Figure 2**). Bidirectionally, zoledronate- or HMBPP-primed monocytes can activate Vγ9Vδ2^+^ T cells through phosphoantigen accumulation and presentation, leading to γδ T-cell proliferation and bacterial pathogen killing (76, 263). However, *in vitro*, it has also been reported that in the presence of zoledronate, monocytes and Vδ2^+^ γδ T cells can negatively regulate each other by inducing apoptosis (266, 267). It is interesting to note that the contact-dependent stimulation of Vγ9Vδ2^+^ T cells by monocytes *via* the intercellular adhesion molecule 1 (ICAM-1)/LFA-1 engagement can be disrupted by blocking CD11a with monoclonal antibody (78). In contrast to these *in vitro* results, *in vivo* treatment with zoledronate or other aminobisphosphonates has shown varying effects, with some studies reporting an increase in circulating monocyte numbers, while others found no difference (77, 268). This suggests that the relationship between these cells may be more nuanced and context-dependent than first thought and will require further investigation.

The crosstalk between γδ T cells and macrophages has not yet been thoroughly elucidated; however, the effects are again cell subtype- and context-dependent. Macrophages have been demonstrated to recruit Vγ9Vδ2^+^ T cells to the site of infection *via* IP-10 and CXCR3 receptor–ligand interactions (80). Once this occurs, Vδ2^+^ γδ T cells can drive the local cytotoxic response *via* granzyme and perforin release or Fas ligand binding (79, 81, 82). Both Vδ1^+^ cells and Vδ2^+^ cells have been shown to produce CCL3, CCL4 (MIP-1α and MIP-1β), and CXCL10, which find their respective cognate receptors expressed by macrophages (70, 138). *In vitro*, the supernatant of cultured γδ T cells has been shown to induce macrophage activation *via* IFN-γ, TNF-α, and GM-CSF production, arguing for a tightly regulated and balanced interplay between these immune cell populations (265). This was further demonstrated by studies showing that IFN-γ and TNF-α released by activated Vγ9Vδ2^+^ T cells can induce cyclooxygenase-2 (COX2) expression and prostaglandin E2 (PGE2) release by both macrophages (**Figure 2**) and tumor cells, and this downregulates the cytotoxic response of γδ T cells (269, 270) and plays a major role in tumor immune escape (271, 272). Furthermore, galectin-9 on both γδ T cells and pancreatic tumor cells has been shown to bind dectin-1 on tumor-infiltrating macrophages, leading to M2 macrophage polarization and subsequent downregulation of IFN-γ and TNF-α production by γδ T cells (273, 274).

## Crosstalk between γδ T cells and neutrophils

Neutrophils are another immune cell population with complex interactions with γδ T cells at peripheral sites of inflammation and in the TME. Zoledronate-activated Vγ9Vδ2^+^ T cells release cytokines and chemokines such as IFN-γ, TNF-α, IL-6, and MCP-2, and these have been demonstrated *in vitro* to induce neutrophil migration, activation, phagocytosis, degranulation, and release of α-defensins (83). In a differing context using a bacterial phosphoantigen, HMBPP-activated Vγ9Vδ2^+^ T cells produce CXCL8 and TNF-α, which together mediate neutrophil recruitment, induce CD11b upregulation and prevent apoptosis, and downregulate CD62L, allowing neutrophil adhesion (78). This finding was further corroborated by Sabbione et al., showing that HMBPP-activated Vδ2^+^ γδ T cells can stimulate CD11b expression and myeloperoxidase production by neutrophils (86), all of which imply a stimulatory role of γδ T cells towards these granulocytes. In another study, tissue-resident Vδ1^+^ γδ T cells were shown to regulate the recruitment of neutrophils to the site of bacterial infection *via* IL-17 secretion (275). In the absence of Vδ1^+^ γδ T cells, the production of IL-17 is reduced and leads to lower numbers of neutrophil recruitment to the site of infection (275).

Interestingly, activated neutrophils can inhibit CD25 and CD69 expression, IFN-γ production, and cell proliferation of Vδ2^+^ γδ T cells either spontaneously or in response to HMBPP (86). This is dependent on initial TNF-α production by γδ T cells, which then induces reactive oxygen species (ROS) secretion from neutrophils (86) (**Figure 2**). These processes can be independent of cell–cell contact; however, the inhibition is more potent if cells are allowed to interact and form conjugates (86). Neutrophils can take up zoledronate, and despite also expressing BTN2A1 and BTN3A1, they do not have the capability of activating Vγ9Vδ2^+^ T cells, which may be due to their extremely limited production and accumulation of IPP (276–278). Rather, these zoledronate-activated neutrophils inhibit TNF-α and IFN-γ production and proliferation of Vγ9Vδ2^+^ T cells *via* ROS, arginase-1, and serine protease production. Some serine proteases are also able to downregulate BTN3A1 expression on PBMCs, which has downstream consequences for BTN-mediated activation of Vδ2^+^ γδ T cells (84, 85). Furthermore, Vδ1^+^ γδ T cells have been shown to exhibit reduced proliferation in the presence of hydrogen peroxide as well as decreased glutathione production, which may be indicative of ROS-dependent neutrophil inhibition (139). In some instances, however, neutrophils that have phagocytosed HMBPP-producing bacteria subsequently release HMBPP, which is then able to activate Vγ9Vδ2^+^ T cells. This results in CD25, CD69, LFA-1, IFN-γ, and TNF-α production and is crucial for initiating an immediate anti-inflammatory response (78).

Functionally, pancreatic tumor cell killing by γδ T cells within a PBMC context is decreased in the presence of neutrophils, in both unstimulated and zoledronate-activated conditions (279). However, when pancreatic tumor cells are co-cultured with purified, expanded γδ T cells and neutrophils, tumor cell lysis is increased compared to co-culture with γδ T cells alone, which can be attributed to elevated granzyme B and IFN-γ production. These conflicting observations may be explained by differences in immune cell subpopulation crosstalk within PBMCs, or by differing polarization of neutrophils: N1 neutrophils are tumor suppressive while N2 neutrophils have a pro-tumoral phenotype (280). It is worth noting that a higher neutrophil-to-lymphocyte ratio in a cohort study of 1,714 cancer patients treated with immune checkpoint inhibitors was recently reported to significantly correlate with low progression-free survival, poor response rates, and low clinical benefit (281). Considering the immunosuppressive functions of activated neutrophils on γδ T-cell activation as discussed above, this may partly contribute to the poor outcomes in cancer patients with higher neutrophil-to-lymphocyte ratios.

## Crosstalk between γδ T cells and dendritic cells

DCs are professional APCs, and consist of classical or conventional DCs (cDCs), including cDC1 (CD11c^+^ and CD141^+^) and cDC2 (CD11c^+^ and CD1c^+^), and plasmacytoid DCs (pDCs, CD11c^-^, CD123^+^, and CD303^+^) (282, 283). Their key role in anti-tumor immunity is well described, but the interactions between DCs and γδ T cells is lacking behind. It has been shown that upon recognition of bacteria-infected or tumor cells, activated Vγ9Vδ2^+^ T cells can aid DC maturation through cytokine secretion (IFN-γ and TNF-α) (87, 88), and promote maturation of antigen-expressing immature DCs (monocyte-derived) in circulation *via* contact-dependent mechanisms (Fas/FasL, CD40/CD40L, and TCR/CD1) independent from TLR signaling (89–91, 93) (**Figure 2**). These Vγ9Vδ2^+^ T cell-matured DCs upregulate HLA-DR, CD25, CD40, CD80, CD83, and CD86, and are capable of cytokine production (TNF-α, IL-12, and IL-15, but not IL-10), antigen presentation, and stimulation of naïve CD4^+^ αβ T cells (76, 87, 89, 92, 284–288). In addition, Vγ9Vδ2^+^ T cell-derived cytokines (IFN-γ and TNF-α) can also enhance TLR-dependent DC maturation, upregulate CCR7 (lymph node-homing receptor), and facilitate their migration to lymphoid tissues for CD4^+^ αβ T-cell priming (289, 290).

In contrast, the tumor-derived chemokine ligand CXCL10 can promote the expansion of Vδ1^+^ γδ Treg cells that infiltrate solid tumors and induce immune senescence in DCs, and prevent DC maturation (by inhibiting CD80, CD83, CD86, and HLA-DR expression), DC function (decreased IL-6 and IL-12 production), and DC phenotype (inability to stimulate naïve T-cell proliferation) *via* the TLR8 signaling pathway or by killing of DCs through a perforin-mediated pathway (102–107) (**Figure 2**).

In turn, DCs can mediate Vγ9Vδ2^+^ T-cell activation by sensing/presenting HMBPP and induce γδ T-cell proliferation in the presence of IL-2, IL-15, and IL-21 (76, 94–96). Immature DCs can enhance the ability of Vγ9Vδ2^+^ T cells to secrete inflammatory cytokines necessary for γδ T-cell maturation (TNF-α) in part due to the ability of DCs to upregulate and/or sense phosphoantigens (88). Mature cDCs and pDCs (monocyte-derived) can secrete cytokines (IL-1β, IL-12, IL-18, IFN-γ, and TNF-α) that activate Vγ9Vδ2^+^ T cells, enhancing their proliferation and cytotoxic function (IL-18-mediated cytotoxicity against tumor cells) (287, 291–296). In the presence of phosphoantigen, IL-15-producing DCs (monocyte-derived) can also activate γδ T cells in a contact-dependent manner (CD86) and induce secretion of IFN-γ (284, 297, 298). Zoledronate-treated immature and mature DCs (monocyte-derived) can induce phosphoantigen-mediated activation and expansion of effector Vγ9Vδ2^+^ T cells capable of co-stimulatory and cytotoxic functions *via* the expression of CD40L (299–303).

In summary, different γδ T-cell subsets can either aid and promote or inhibit DC maturation and function (7, 13, 304, 305), while DCs can activate and expand Vγ9Vδ2^+^ T cells (7, 13, 304–307). The crosstalk between γδ T cells and DCs can thus have downstream anti- or pro-tumoral effects with therapeutic potential, albeit warranting further investigation using DCs that are not monocyte-derived (8, 150, 308).

## Outlook and future perspective

Our understanding on γδ T cells continues to expand and their contributions in bridging the innate and adaptive anti-tumor immune responses are becoming more evident. Multiple studies are now highlighting their role in interacting with and orchestrating a variety of other immune cell subsets as reviewed here. Traditionally, γδ T cell-based cancer immunotherapies have been focused on assessing the efficacy of activated γδ T cells alone in mediating tumor clearance (41–46, 145, 157, 163, 165, 309). Although these past clinical trials have shown that γδ T cell-based immunotherapies were safe and well tolerated in patients, given the limited success to date (8, 10, 19, 101, 310–312), more innovative strategies aiming to overcome the challenges and immunosuppression within the TME should be thoroughly explored. Notably, with the ever-increasing numbers of studies demonstrating the intricate network of immune interactions within the TME, it is high time to deeply explore some of these interactions and to gain valuable insights into the unique immunomodulatory functions of γδ T cells in the context of cancer immunotherapy. Such acquired knowledge can be fully harnessed to develop a multipronged γδ T cell-based immunotherapy focusing on γδ T cells’ capability to influence the activities of other tumor-infiltrating immune cells *via* rapid cytokine and chemokine secretion, expression of various co-stimulatory molecules, and the professional APC functions in cross-priming and presenting antigens to αβ T cells.

For example, we are now armed with several potent therapeutic agents including the agonist antibodies against BTN3A1 (clone 20.1, CTX-2026, and ICT-01) and BTN2A1 (ICT-0302) that are capable of activating and enhancing the immunomodulatory functions of Vγ9Vδ2^+^ T cells (48–53, 227, 313, 314). Treatment targeting BTN3A1 (CTX-2026) can induce coordinated Vγ9Vδ2^+^ and αβ T-cell responses for tumor cell killing and represents a promising therapeutic approach that could be combined with other immune checkpoint inhibitors targeting PD-1/PD-L1 (nivolumab and pembrolizumab), CTLA-4/CD86 (ipilimumab and tremelimumab), KIRs (lirilumab and IPH4102), ILT-2 (anti-ILT-2, anti-HLA-G1, anti-FasL), and NKG2A (monalizumab) to circumvent potential immunosuppression in TME (11, 204, 205). These anti-tumor responses could potentially be further enhanced by inducing the expression of NKG2D ligands in tumor cells using a proteasome inhibitor (bortezomib) and an alkylating agent (temozolomide) to promote orchestrated NKG2D-mediated tumor cell lysis by tumor-infiltrating NK, CD8^+^ αβ, and γδ T cells (240, 256, 257). Moreover, CD137 (4-1BB) co-stimulation with recombinant human CD137L can boost the therapeutic effect of Vγ9Vδ2^+^ T cell-based immunotherapy and lead to heightened NK cell-mediated cytotoxicity (73, 75, 101, 315). Taken together, such combined therapeutic treatment will be a powerful approach to elicit concerted anti-tumor responses in different tumor-infiltrating immune cells and help to maximize the efficacy of future γδ T cell-based immunotherapy treatments in cancer patients.

## Author Contributions

KFC, JDGD, SO, and AB wrote and prepared the manuscript draft. KFC prepared the figures. All authors contributed to the article and approved the submitted version.

## Funding

AB is supported by a fellowship from the DHHS acting through the Victorian Cancer Agency. KFC is awarded the ECRs and MCRs Awards (ABC scheme 2022-2023) funding from La Trobe University (WBS: 3.2515.01.20). JDGD was supported by Cure Cancer Australia through the Cancer Australia Priority—Driven Cancer Research Scheme (#1187815). The contents of the published material are solely the responsibility of La Trobe University and do not reflect the views of Cancer Australia.

## Conflict of Interest

AB declares research funding from CSL Ltd. AB and SO are inventors on a patent about mechanisms to activate γδ T cells.

The remaining authors declare that the research was conducted in the absence of any commercial or financial relationships that could be construed as a potential conflict of interest.

## Publisher’s Note

All claims expressed in this article are solely those of the authors and do not necessarily represent those of their affiliated organizations, or those of the publisher, the editors and the reviewers. Any product that may be evaluated in this article, or claim that may be made by its manufacturer, is not guaranteed or endorsed by the publisher.
